# Identifying the Quantitative Trait Locus and Candidate Genes of Traits Related to Milling Quality in Rice via a Genome-Wide Association Study

**DOI:** 10.3390/plants13101324

**Published:** 2024-05-11

**Authors:** Changmin Hu, Xinru Li, Mengyuan Zhang, Chunyu Jing, Mei Hai, Jiaming Shen, Qing Xu, Xiaojing Dang, Yingyao Shi, Erbao Liu, Jianhua Jiang

**Affiliations:** 1 College of Agronomy, Anhui Agricultural University, Hefei 230036, China; 2 Institute of Rice Research, Anhui Academy of Agricultural Sciences, Hefei 230031, China

**Keywords:** rice, milling quality, genome-wide association study, quantitative trait loci, candidate gene

## Abstract

Milling quality directly affects production efficiency in rice, which is closely related to the brown rice recovery (BRR), the milled rice recovery (MRR) and the head milled rice recovery (HMRR). The present study investigated these three traits in 173 germplasms in two environments, finding abundant phenotypic variation. Three QTLs for BRR, two for MRR, and three for HMRR were identified in a genome-wide association study, five of these were identified in previously reported QTLs and three were newly identified. By combining the linkage disequilibrium (LD) analyses, the candidate gene *LOC_Os05g08350* was identified. It had two haplotypes with significant differences and Hap 2 increased the BRR by 4.40%. The results of the qRT-PCR showed that the expression of *LOC_Os05g08350* in small-BRR accessions was significantly higher than that in large-BRR accessions at Stages 4–5 of young panicle development, reaching the maximum value at Stage 5. The increase in thickness of the spikelet hulls of the accession carrying *LOC_Os05g08350*^TT^ occurred due to an increase in the cell width and the cell numbers in cross-sections of spikelet hulls. These results help to further clarify the molecular genetic mechanism of milling-quality-related traits and provide genetic germplasm materials for high-quality breeding in rice.

## 1. Introduction

As a major cereal crop, rice (*Oryza sativa* L.) is crucial to the food security of more than half of the world’s population. With the improvement in people’s living standards, the demand for rice quality is increasing. Improving rice quality while maintaining the current high yields, is one of the main goals of rice breeding [1,2]. As an important indicator of rice quality, the milling quality directly determines the final yield and the broken kernel recovery of the milled rice. Usually, the brown rice recovery (BRR), milled rice recovery (MRR), and head milled rice recovery (HMRR) are used to measure the milling quality. Among them, the HMRR is an important index of milling quality, which directly determines the appearance and commodity value of rice [2]. Therefore, mining the major quantitative trait loci (QTLs) for milling quality will help to elucidate the molecular regulation mechanism of rice quality and provide valuable genetic resources for the breeding of high-quality rice.

Studies have shown that BRR, MRR, and HMRR are quantitative traits controlled by multiple genes and are susceptible to environmental influences [3,4,5]. Many researchers have conducted extensive genetic mapping studies on processing quality. To date, 74 QTLs for BRR, 62 for MRR, and 57 for HMRR have been identified; these QTLs are distributed on all 12 chromosomes (Table S1) [6,7,8,9,10,11,12,13,14,15,16,17,18,19,20,21,22,23,24,25]. The phenotypic variation explained (PVE) ranged from 7.30% to 42.5% for BRR, 11.3% to 44.8% for MRR, and 4.60% to 45.4% for HMRR. Among these QTLs, only a few major ones were finely mapped. Ren et al. [21] mapped a major QTL *qBRR-10* on chromosome 10, which was finely mapped to a 39.5 kb region containing six candidate genes. Most of these QTLs were detected in F_2_ backcross inbred-line populations; doubled-haploid, recombinant-inbred-line populations; and chromosome-segment-substitution-line populations, and only a few were identified in natural populations. The elite alleles detected in the former populations are limited, and it is difficult to utilize them in rice breeding. Therefore, to improve the milling quality of rice, it was necessary to identify germplasm resources and discover more QTLs/genes for milling quality by conducting a genome-wide association study (GWAS) in a natural population. 

In the present study, the phenotype values of BRR, MRR, and HMRR in 173 rice germplasms were investigated and a GWAS was carried out to detect QTLs. Then, the candidate genes were predicted. These results can provide a material foundation for improving the milling quality of rice and a molecular foundation for elucidating the genetic mechanism of milling quality. 

## 2. Results

### 2.1. Phenotypic Evaluation

**Figure 1:**
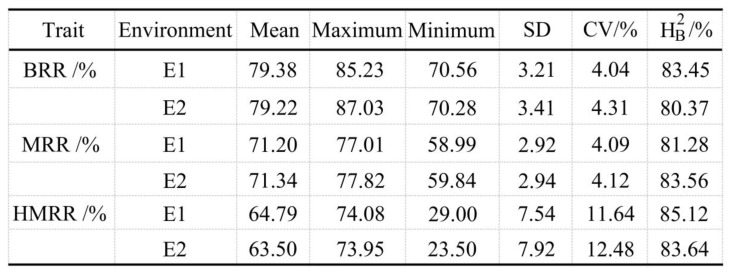
Phenotypic statistics of milling-quality-related traits in E1 and E2. H^2^_B_, broad-sense heritability; BRR, brown rice recovery; MRR, milled rice recovery; HMRR, head milled rice recovery.

**Figure 2:**
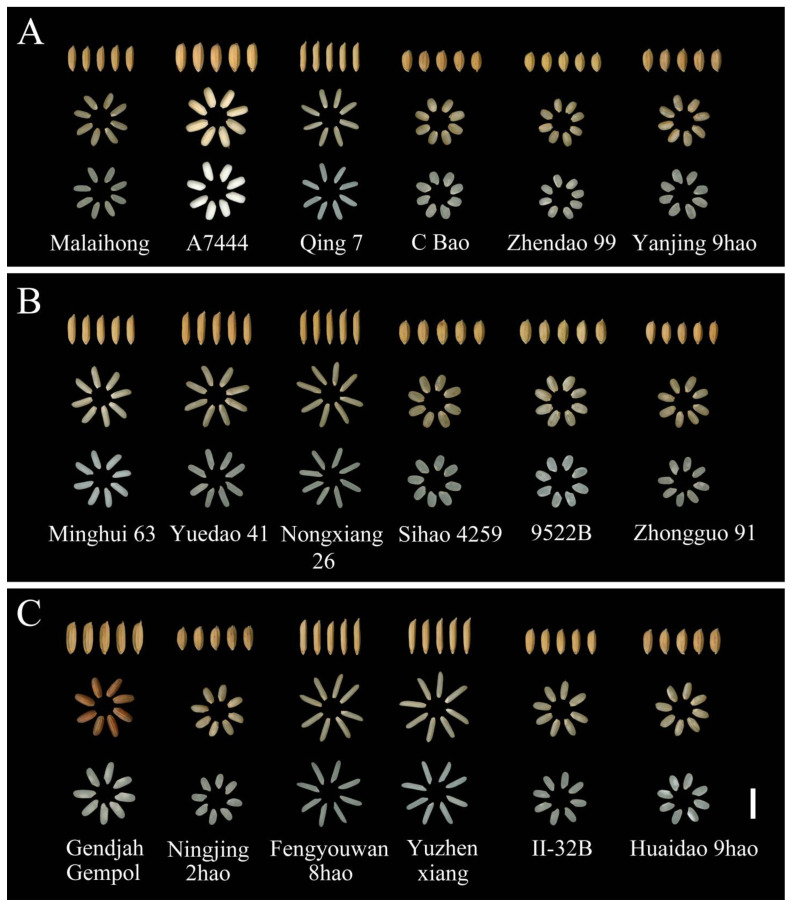
The performance of rough rice, brown rice, and milled rice in different materials. (**A**) The performance of rough rice, brown rice, and milled rice in part-rice materials with different BRRs. (**B**) The performance of rough rice, brown rice, and milled rice in part-rice materials with different MRRs. (**C**) The performance of rough rice, brown rice, and milled rice in part-rice materials with different HMRRs. BRR, brown rice recovery; MRR, milled rice recovery; HMRR, head milled rice recovery.

For the BRR trait, the maximum value was 85.23% in E1 and 87.03% in E2 (Figure 1). The minimum value was 70.42% across the two environments. The heritability in a broad sense (H^2^_B_) was more than 80% across the two environments. Figure 2A shows the un-hulled rice, brown rice, and milled rice parts of rice materials with different BRR values. The material Malaihong had the minimum BRR (70.52%) and the material Yanjing 9hao had the maximum BRR (86.13%). 

For the MRR trait, the mean value was 71.20% in E1 and 71.34% in E2 (Figure 1); the coefficient of variation (CV) ranged from 4.09% to 4.12%. The H^2^_B_ was 81.28% in E1 and 83.56% in E2. Figure 2B shows the un-hulled rice, brown rice, and milled rice parts of the rice materials with different MRR values. The material Minghui 63 had the minimum MRR (59.42%) and the material Zhongguo 91 had the maximum MRR (77.42%). 

For the HMRR trait, the mean value was 64.79% in E1 and 63.50% in E2 (Figure 1). The minimum value was 29.00% in E1 and 23.50% in E2, and the CV was 12.06% across the two environments. The H^2^_B_ was 85.12% in E1 and 83.64% in E2. Figure 2C shows the un-hulled rice, brown rice, and milled rice parts of the rice materials with different HMRR values. The material Gendjah Gempol had the minimum HMRR (27.35%) and the material Huaidao 9hao had the maximum HMRR (76.36%). The results of two-way analysis of variance (ANOVA) showed that there were significant differences among the genotypes for the BRR, MRR, and HMRR, and no significant differences between the two environments for BRR and HMRR (Table S2). These results suggest that the traits of BRR, MRR, and HMRR are mainly controlled by genotypes, which can be stably inherited in the two environments.

### 2.2. Identification of QTLs for Traits Related to Rice Milling Quality via a GWAS

**Figure 3:**
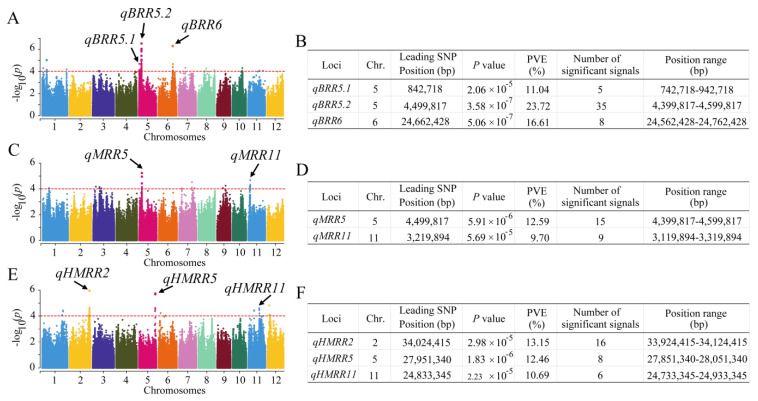
Identification of QTLs in rice via GWAS. (**A**) Manhattan plots for BRR in the whole population of rice accessions. (**B**) Information related to the identified QTLs for BRR. (**C**) Manhattan plots for MRR in the whole population of rice accessions. (**D**) Information related to the identified QTLs for MRR. (**E**) Manhattan plots for HMRR in the whole population of rice accessions. (**F**) Information related to the identified QTLs for HMRR. The Y axis shows negative log10-transformed *p*-values, and dots above the red line show the significant SNPs in the QTL region. The black arrows indicate the identified QTL. BRR, brown rice recovery; MRR, milled rice recovery; HMRR, head milled rice recovery; PVE, phenotypic variation explained.

The GWAS was conducted to identify the possible natural variation in traits related to rice milling quality. Manhattan plots were constructed to display the significant SNPs related to rice milling quality. For the BRR trait, a total of three QTLs were identified, which were located on chromosomes 5 and 6 (Figure 3A,B). There were 35 significant SNPs in the *qBRR5.2* region. The PVE ranged from 11.04% to 23.72%. For the MRR trait, we identified two QTLs located on chromosomes 5 and 11 (Figure 3C,D), with PVEs of 12.59% and 9.70%, respectively. For HMRR, three QTLs were identified, which were located on chromosomes 2, 5, and 11 (Figure 3E,F). The PVE ranged from 10.69% to 13.15%. Among them, the largest number of significant SNPs (16) existed in the *qHMRR2* region on chromosome 2. The QTL region of *qBRR5.2* was the same as that of *qMRR5*. Meanwhile, the QTL *qBRR5.2* possessed the largest number of significant SNPs (35), and the greatest PVE (23.72%) and −log_10_(*p*) (6.45), so it was considered the major QTL. As such, we focused our further investigations on *qBRR5.2*. 

### 2.3. Identification of Candidate Genes

**Figure 4:**
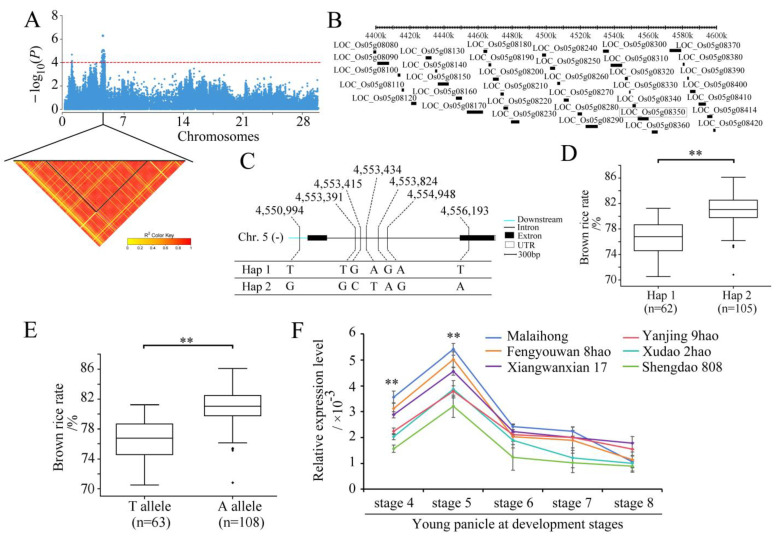
Identification of the candidate genes for the QTL *qBRR5.2* of BRR. (**A**) Manhattan plots for BRR on the chromosome 5 and the identification of the haplotype block of *qBRR5.2*. The Y axis shows negative log10-transformed *p*-values, and the dots above the red line show the significant SNPs in the QTL region. Pairwise LD was determined via the calculation of *r*^2^ (the square of the correlation coefficient between SNP states). (**B**) Identification of candidate genes in the region of *qBRR5.2*. (**C**) Haplotypes of *LOC_Os05g08350* associated with BRR in rice. (**D**,**E**) Boxplots of BRR in accessions containing the different haplotypes (**D**) and elite alleles (**E**). (**F**) Expression analysis of the candidate gene *LOC_Os05g08350* in different materials at different stages in young panicles. The relative expression values were normalized to the rice UBQ gene. Error bars indicate the standard deviation, and asterisks indicate significant differences determined using Student’s *t*-test (** *p* < 0.01).

The candidate genes containing *qBRR5.2* on chromosome 5 were analyzed (Figure 4A). In total, 36 positional candidate genes were identified for *qBRR5.2* (Figure 4B). Based on the LD analysis, the LD block region was determined to be the 4,430,827–4,571,761 bp region, which contained 13 candidate genes. Based on SNP information, 10 of the 13 genes contained nonsynonymous SNPs (Tables S3 and S4). However, only one nonsynonymous SNP was significantly associated with BRR according to the GWAS (Table S3); it was located within the gene locus *LOC_Os05g08350*. The full length of *LOC_Os05g08350* was 5810 bp, and included two exons and one intron (Figure 4C). The gene *LOC_Os05g08350* encoded an F-box domain containing proteins with 554 amino acids. The SNP polymorphisms occurred in the downstream, intron, and coding sequences of the gene. This resulted in two haplotypes being identified (Figure 4C). The haplotype Hap 1 was associated with a smaller BRR, while the haplotype Hap 2 was associated with a larger BRR (Figure 4D). The SNP site (4,556,193) caused a change from base A to base T at nt 1357 in the cDNA sequence, resulting in an amino acid change from cysteine (C) to stop-gained at amino acid 272. The average BRR values of the 63 accessions carrying the TT allele was 76.50 ± 8.60%, while those with the AA allele had an BRR of 80.90 ± 4.20%. There were highly significant differences between the TT allele and the AA allele at *p* < 0.01 (Figure 4E).

The relative expression levels of *LOC_Os05g08350* in the spikelet between three accessions, each with a high or low BRR, were analyzed at different young panicle development stages via qRT-PCR. The expression of the TT allele in each of the three accessions (Malaihong, Fengyouwan 8hao, and Xiangwanxian 17) with a small BRR was significantly higher than that of the AA allele in each of the three accessions (Yanjing 9hao, Xudao 2hao, and Shendao 808) with a large BRR (Figure 4F). The expression of *LOC_Os05g08350* in small-BRR accessions was significantly higher (*p* < 0.01) than that in large-BRR accessions at Stages 4 and 5 of young panicle development, reaching a maximum value at Stage 5 (Figure 4F). Moreover, there was no significant difference between small-BRR accessions and large-BRR accessions from Stage 6 to Stage 8 of young panicle development (Figure 4F). These results suggest that the reduced expression of the AA allele may increase the BRR. 

### 2.4. Evolutionary Analysis of LOC_Os05g08350 in Gramineous Species

**Figure 5:**
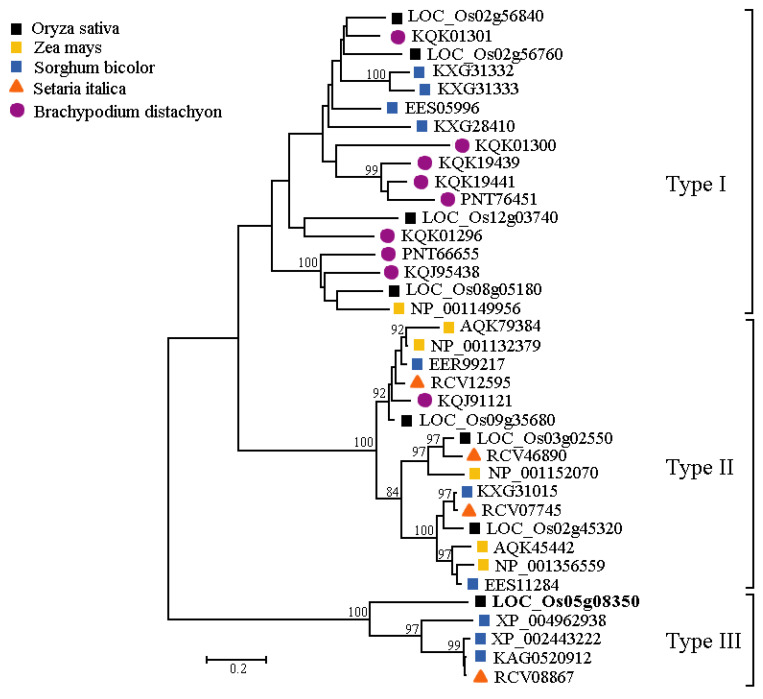
Phylogenetic analysis of the LOC_Os05g08350 protein. Phylogenetic tree of the complete amino acid sequence from different gramineous plants (*Oryza sativa*, *Zea mays*, *Sorghum bicolor*, *Setaria italica*, and *Brachypodium distachyon*).

In the protein sequence encoded by *LOC_Os05g08350*, there is an F-box domain at the N-terminal. The SCF complex is an important E3 ubiquitin ligase. The F-box domain can interact with the subunit SKP1 of the SCF complex to achieve the specific degradation of different substrates [26]. In order to clarify the evolution of the F-box gene family in gramineous plants, the homologous proteins of the F-box gene family in five representative gramineous plants were further researched. The five representative gramineous plants were *Oryza sativa*, *Zea mays*, *Sorghum bicolor*, *Setaria italica*, and *Brachypodium distachyon*. The locus, chromosome, protein length, and protein-position information are listed in Table S5. The phylogenetic tree was constructed from 37 members of the gene families (Figure 5). The F-box gene family can be divided into three independent types in gramineous plants. Here, we refer to these three types as Type I, Type II, and Type III. There are 17 members in the Type I group, 15 in Type II, and 5 in Type III. In rice, there are four members in Type I, three in Type II, and one in Type III. The LOC_Os05g08350 protein in this study was a Type III protein. 

### 2.5. Protein Structure Analysis of LOC_Os05g08350

**Figure 6:**
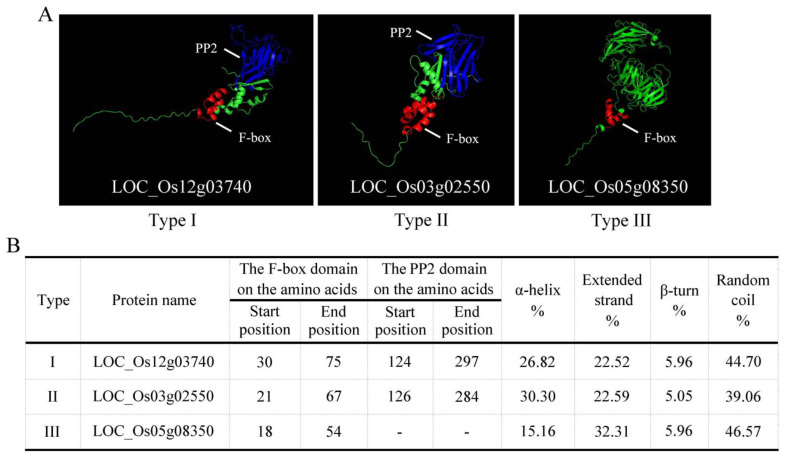
The three types of protein tertiary structure and basic information statistics. (**A**) The three types of protein tertiary structure. The shape of the tertiary structure of proteins and the location of the key domains can be observed. (**B**) Basic statistics related to the protein’s secondary structure. PP2, phloem protein 2.

In order to further clarify the differences in the three types of F-box domains from the tertiary structure of the proteins, we carried out a modeling analysis on the Type I-LOC_Os12g03740, the Type II-LOC_Os03g02550, and the Type III-LOC_Os05g08350. The tertiary structures of the three types of F-box proteins are shown in Figure 6A. The Type I-LOC_Os12g03740 and Type II-LOC_Os03g02550 have not only F-box domains, but also PP2 domains (Figure 6A). The Type III-LOC_Os05g08350 contains only the F-box domain, and the protein structure is approximately divided into two parts, with a large space in the middle (Figure 6A). Meanwhile, there are significant difference in the α-helices and extended strands, but there are no differences in the β-turns and random coils among the three types (Figure 6B). These results provide an additional basis for the classification of F-box proteins in different types in rice. 

### 2.6. LOC_Os05g08350^TT^ Increased the Cell Numbers and Cell Widths in Cross-Sections in Spikelet Glumes

**Figure 7:**
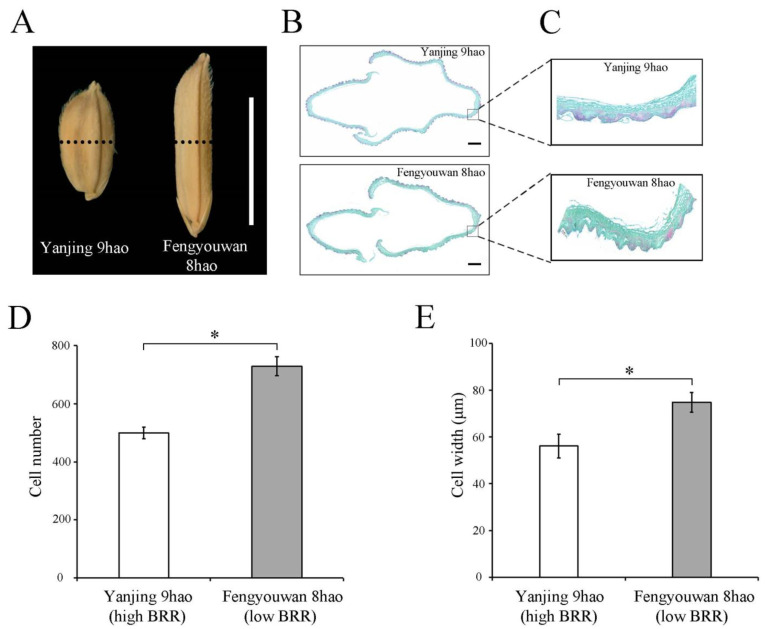
Histological analyses of spikelet hulls. (**A**) Spikelets at maturity. Scale bar, 1 cm. (**B**) Cross-section of spikelet hulls. Scale bar, 200 μm. A Leica microtome was used to cut the spikelet hulls. (**C**) Magnified view of the spikelet hull cross-sections boxed in B. (**D**) Total cell numbers from the inside to the outside of parenchymal cell layers of spikelet hulls. (**E**) Cell widths from the inside to the outside of parenchymal cell layers of the spikelet hulls. The values shown are the mean ± SD. Student’s *t*-test was used for comparisons (n = 10 spikelets, * *p* < 0.05).

To determine whether the thickness of spikelet hulls affects BRR, we compared the cell numbers and cell widths in spikelet-hull cross sections of Yanjing 9hao with a high BRR value and Fengyouwan 8hao with a low BRR value. There were significant differences in the cell numbers and cell widths from the inner to outer parenchymal cell layers of the spikelet hulls between Yanjing 9hao and Fengyouwan 8hao (Figure 7). These findings suggest that the increase in the thickness of the spikelet hulls in Fengyouwan 8hao is due to an increase in the widths and numbers of cells in a cross-section in spikelet hulls. 

## 3. Discussion

The milling quality of rice is a quantitative trait controlled by multi-genes, it is influenced by the environment, water content, and processing methods. Among these factors, genetic factors are the most important. There were significant differences in the milling quality among different germplasm resources. The ranges of the BRR and MRR were 70.28–87.03% and 58.99–77.82%, respectively (Figure 1). The minimum value of the HMRR was 23.50%, the maximum value was 74.08%, and the CV of HMRR was 11.64–12.48% (Figure 1). These results indicate that the population examined in this study exhibited abundant phenotypic variation, providing a material basis for the discovery of favorable alleles for traits related to milling quality. Materials with high BRR, MRR, and HMRR values may exist in different grain sizes (Figure 2), which is consistent with the viewpoint reported by Fang et al. [23]. Therefore, it is possible to select long-grain individuals with high BRR, MRR, and HMRR values in the progeny population by selecting suitable parents. 

Three QTLs for BRR, two for MRR, and three for HMRR were detected in this study (Figure 3). Based on the Gramene website (http://www.gramene.org/markers/, accessed on 14 March 2021), BLAST (http://blast.ncbi.nlm.nih.gov/Blast.cgi, accessed on 20 May 2022), and the China Rice Data Center database (http://www.ricedata.cn/gene/list/1499.htm, accessed on 15 August 2022), these QTLs were compared with those reported previously. The position range of QTL *qBRR5.1* for BRR overlapped with the flanking region (264,087–1,861,366 bp) reported by Weng et al. [12] (Table S6). The QTL *qMRR11* was in the same region as the QTL *qMR-11* reported by Weng et al. [12] (Table S6). The QTL *qHMRR2* overlapped with the flanking region (32,774,365–35,272,373 bp) reported by Hu et al. [14] (Table S6). The QTL *qHMRR5* was located in the chromosomal regions where the QTLs for the HMRR were identified by Tan et al. [6], Aluko et al. [9], and Weng et al. [12] (Table S6). The QTL *qHMRR11* overlapped with the flanking region (19,840,132–28,281,693 bp) reported by Yao et al. [25] (Table S6). The remaining QTLs *qBRR5.2*, *qBRR6*, and *qMRR5* were newly identified in this study. 

The major QTLs for milling-quality-related traits were detected in the same chromosome intervals as those previously reported in different populations and environments, indicating that these QTLs exist independently of genetic background and environment. Some studies have reported that there are different degrees of correlation between the appearance quality and milling quality of rice [23]. However, the genes reported to be associated with appearance quality, such as *GW2* [27,28], *GW5* [29,30], *GL6* [31], *GW6* [32], *chalk5* [33], and *OsGIF1* [34], were not detected in this study. This result may be related to the genetic susceptibility of quantitative traits to environmental influences or to the different experimental materials used in other studies. 

The QTL *qBRR5.2*, which is co-located with *qMRR5*, had the largest PVE (23.72%) and the largest number of significant SNP loci (35). In the LD block region of 4,430,827–4,571,761 bp of chromosome 5, gene *LOC_Os05g08350* is a newly identified candidate gene (Figure 4). The full length of *LOC_Os05g08350* is 5810 bp, including two exons and one intron. Gene *LOC_Os05g08350* encodes a protein of 554 amino acids. The base T-to-A nonsynonymous mutation in the cDNA sequence of *LOC_Os05g08350* caused the high BRR phenotype (Figure 4). Based on the haplotype classification, the average BRR of Hap 1 and Hap 2 was 76.5% and 80.9%, respectively. Hap 2 can increase the brown rice recovery by 4.4%. These results suggest that the gene *LOC_Os05g08350* has potential application value in improving the BRR via molecular breeding. 

The qRT-PCR results showed that the relative expression levels in the accessions with large-BRR-carrying AA alleles were significantly lower than those in the accessions with small-BRR-carrying TT alleles (Figure 4F). The gene *LOC_Os05g08350* encodes a OsFBX162-F-box domain containing protein. Baute et al. [35] found that the F-box protein FBX92 affects cell division leading to different cell numbers that ultimately affect leaf size. Thus, we speculated that in the process of spikelet hull formation, the low expression of gene *LOC_Os05g08350* reduced the cell division of spikelet hulls and caused the spikelet hulls to become thinner, resulting in a large BRR. On the contrary, the high expression of the gene *LOC_Os05g08350* increased the cell division of the spikelet hull, which caused the spikelet hull to become thicker, resulting in a small BRR. The histological analysis revealed that *LOC_Os05g08350* regulated the cell width and the number of cross-sections in the spikelet glumes, which confirmed this hypothesis. 

The results of the evolutionary analysis showed that LOC_Os05g08350 and other rice F-box genes were divided into different classes. The protein structure of LOC_Os05g08350 is different from that of other rice genes, which may be the main reason why they are classified into different classes and perform different functions. Before now, some functions of F-box genes had been identified, such as the antioxidant *TAFBA1* gene in wheat [36,37,38], the drought-stress negative regulator *AtPP2-B11* gene in *Arabidopsis thaliana* [39], and the *OsFBX76* gene in rice [40], but the mechanism of the F-box gene is still unclear. At present, what we know about the F-box gene is only the tip of the iceberg for the entire plant F-box gene family. Therefore, further research on the F-box gene family is needed. 

## 4. Material and Methods

### 4.1. Plant Materials

A total of 173 rice accessions comprising 63 indica rice varieties and 110 japonica rice varieties were grown at the Experimental Station of the Anhui Academy of Agricultural Sciences (31°52′ N, 117°17′ E) in 2021 and 2022, respectively. The corresponding material names, origins, and sequence IDs of the 173 accessions are listed in Table S7. When the rice seedlings had grown to four leaves with one heart, the seedlings of each piece of material were transplanted. Each material was planted in 4 rows with 9 plants per row. The distance between individual plants was 20 cm × 20 cm. Each accession was repeated twice in the field with a routine field management. The meteorological data from sowing to harvest of 173 rice materials in two environments are listed in Table S8. Table S9 lists the flowering date and maturity date and corresponding part meteorological data of 173 rice materials in two environments.

### 4.2. Phenotypic Investigation

The harvested seeds were air-dried for three months and then used in the follow-up experiments. For each repetition, 100 g of seeds of each variety was selected and milled into brown rice using a machine (JLGJ4.5, Topp Yunnong Technology Co., Ltd., Hangzhou, China). Next, the brown rice was milled into the milled rice using a mini-type rice machine (JNMJ3, Topp Yunnong Technology Co., Ltd., Hangzhou, China). Then, the weights of the brown rice and milled rice of each variety were determined using an electronic balance. The head milled rice was selected from the milled rice and weighted using the electronic balance. We considered the rice to be the head milled rice when the rice length was more than 4/5 of the average length of all the rice. The BRR, MRR, and HMRR were calculated according to the following formulas: BRR (%) = the weight of brown rice (g)/the weight of rough rice (g) × 100, MRR (%) = the weight of milled rice (g)/the weight of rough rice (g) × 100, and HMRR (%) = the weight of head milled rice (g)/the weight of rough rice (g) × 100. Two repetitions were conducted for each trait’s measurement. The mean value of the two repetitions was calculated as the BRR, MRR, and HMRR percentages of each variety. 

### 4.3. Genotypic Data

The re-sequenced data of the 173 varieties were reported by Dang et al. [41] and deposited in the NCBI Sequence Read Archive with the accession number PRJNA554986. The paired-end sequence reads were aligned using the Nipponbare sequence (IRGSP 1.0) as the reference, with Bowtie 2 software [42]. More than 95% of reads had a mapping score >60, which could be mapped to the Nipponbare reference genome. The SNP calling was carried out using the HaplotypeCaller of GATK 3.8-0 (University of South Florida, Tampa, FL, USA). The missing data in the genotype data were imputed using the Beagle v4.1 software [43]. The SNPs with a minor allele frequency (MAF) >5% and a missing rate <20% were selected, and the total number of SNPs was 1,322,884. 

### 4.4. Genome-Wide Association Analysis

The GWAS was carried out based on the mixed linear model in eXpedited [44]. The TASSEL 5.2.1 software was used to calculate the kinship matrix [45]. According to the method reported by Yang et al. [46], when more than five significantly associated SNPs were detected and there was a peak signal around the leading SNP in the 200 kb region, we considered it to be a QTL region. The 200 kb region contained 100 kb downstream and 100 kb upstream of the leading SNP. The R package “LDheatmap” was used to construct the linkage disequilibrium (LD) heatmap surrounding the peaks [47]. The Manhattan and quantile–quantile plots were drawn using the R package qqman [48]. The genome-wide significant thresholds of the GWAS were determined as 1.0 × 10^−4^ at a nominal level of 0.05 based on the correction method reported by Benjamini and Hochberg [49]. 

### 4.5. Candidate Gene Analysis

The candidate genes within a 200 kb genomic region were predicted according to the Rice Genome Annotation Project MSU7 database (http://rice.plantbiology.Msu.edu, accessed on 10 February 2024). The LD blocks surrounding significant SNPs were determined using Haploview 4.2 software [50]. The causal gene for each locus was determined according to the gene annotation and expression analysis. 

### 4.6. Haplotype Analysis

The haplotypes of the candidate genes were identified using the RiceVarMap database (http://ricevarmap.ncpgr.cn/) and the China Rice Data Center (https://www.ricedata.cn/). Each haplotype should be carried in more than 20 varieties. 

### 4.7. Quantitative Real Time PCR Analysis of Candidate Genes

The spikelets of young panicles at developmental stages 4, 5, 6, 7, and 8 were sampled from the three samples with high BRR values and three samples with low BRR values. The total RNA was extracted using a TianGen Pure Plant Plus Kit (TianGen Biotech Co., Ltd., Beijing, China). The first-strand cDNA was synthesized using a HisScript II Reverse Transcriptase system (Vazyme Biotech Co., Ltd., Nanjing, China). The UBQ rRNA gene was used as the internal control. Real-time quantitative PCR (qRT-PCR) was performed using SYBR Green (Vazyme Biotech Co., Ltd., Nanjing, China) based on the 96-well thermocycler (Roche Applied Science LightCycler 480, https://lifescience.roche.com/). We performed the PCR following the procedure reported by Hu et al. [51]. For each sample, three biological repetitions were conducted. The primers used for the qRT-PCR are listed in Table S10. The transcript levels of gene expression were calculated following the method reported by Livak and Schmittgen [52]. 

### 4.8. Phylogenetic Tree Construction 

The homologous protein sequences of F-box genes in gramineous species were obtained from NCBI (https://www.ncbi.nlm.nih.gov/). DNAMAN 7.0 was used to compare the sequence of homologous proteins. The phylogenetic tree was constructed using the maximum likelihood method in MEGA 5.0 [53]. 

### 4.9. Protein Structure and Modeling

The secondary structure of the protein was analyzed using SOPMA (https://npsa-prabi.ibcp.fr/cgi-bin/npsa_automat.pl?page=npsa_sopma.html, accessed on 18 January 2022). The model was constructed using Alphafold 2 [54] and the model analysis and drawing were performed using PyMOL 3.0 [55]. 

### 4.10. Histological Analysis

Spikelet hulls were fixed in FAA solution (60% (*v*/*v*) ethanol, 5% (*v*/*v*) glacial acetic acid, and 5% (*v*/*v*) formaldehyde). Then, they were put into the plant-softening solution to soften, dehydrated in an ethanol series (30%, 50%, 75%, 85%, 90%, 95%, and 100% (*v*/*v*) ethanol), destained with xylene, and embedded in paraffin. Tissue sections were cut with a Leica microtome (RM2016, Shanghai Leica Instrument Co., Ltd., Shanghai, China). Then the slices were dewaxed, safranin O-fast stained, decolored, fixed-green stained, made transparent, sealed, and observed under a microscope (Nikon Eclipse E100, Nikon Instruments (Shanghai) Co., Ltd., Shanghai, China). 

## Data Availability

Relevant data are included in this paper and its associated Supplementary Materials.

## Supplementary Materials

The following supporting information can be downloaded at: https://www.mdpi.com/article/10.3390/plants13101324/s1, Table S1: A list of previously published QTLs controlling the BRR, MRR, and HMRR, Table S2: The results of the two-way analysis of variance for traits related to rice milling quality, Table S3: The SNP information in the 4.43–4.57 Mb candidate region for BRR, Table S4: Candidate gene annotation in the region 4.43–4.57 Mb associated with the brown rice recovery, Table S5: The base information of F-box genes from five Gramineae plants, Table S6: The QTLs detected in this study overlapped with the QTLs/genes reported previously, Table S7: The information related to the 173 rice accessions used in this study and the mean phenotype value of milling-quality-related traits in the E1 and E2 environments, Table S8: Meteorological data, from sowing to harvest, of 173 rice materials in two environments, Table S9: The flowering date and maturity date and corresponding part meteorological data of 173 rice materials in two environments. Table S10: The sequences of primers used for the qRT-PCR.
