# Supplementary material for: Identifying the Quantitative Trait Locus and Candidate Genes of Traits Related to Milling Quality in Rice via a Genome-Wide Association Study
Source: Plants (Basel). 2024 May 11;13(10):1324. doi: 10.3390/plants13101324 (PMC11124788; doi:10.3390/plants13101324)
Supplement: Supplementary file 1 [file plants-13-01324-s001.zip › Table S1.pdf]

Table S1. A list of previously published QTLs controlling the BRR, MRR, and HMRR.

Traits	Chromosomes												References
	1	2	3	4	5	6	7	8	9	10	11	12	
BRR /%					1								Tan et al. 2001
												1	Mei et al. 2002
	2	1			1								Septiningsih et al. 2003
	1						1	1					Aluko et al. 2004
			1	1					2	1	1		Li et al. 2004
	1		1	1									Liu et al. 2007
	2	1	1	1	1	2		2					Weng et al. 2007
			1										Kepiro et al. 2008
		1	1	1	1								Hu et al. 2011
		1	2		1	1	1	1		1			Mei et al. 2012
	1	2	1	2									Zhang et al. 2013
						1			2				Zhou et al. 2013
		2	1	1			1						Qiu et al. 2015
				1							2		Li et al. 2016
	1							1	1	1			Ren et al. 2016
	1												Zhang et al. 2016
	2		1				1		1		1		Wang et al. 2017
				2		1					1		Fang et al. 2018
	1												Yao et al. 2023
MRR /%			1		1								Tan et al. 2001
										1			Septiningsih et al. 2003
					1		1						Aluko et al. 2004
				1					2	2	1		Li et al. 2004
	2	1	1	1	2	1	1	1	1	1	1	1	Weng et al. 2007
		1	1										Kepiro et al. 2008
				1	1	2				1			Hu et al. 2011
					1	1							Mei et al. 2012
	1	1	1										Zhang et al. 2013
	1	1	1				1				1		Qiu et al. 2015
				1			1	1	1		2		Li et al. 2016
	2	2	1		1	3	1	1	1				Fang et al. 2018
		1					1						Yao et al. 2023

HMRR /%			1										Tan et al. 2001
		1		1		1	1						Mei et al. 2002
	1	1			1								Septiningsih et al. 2003
	1		1			1		1					Aluko et al. 2004
			1			1	1						Li et al. 2004
	1												Liu et al. 2007
		1	1			1	1	1	1				Weng et al. 2007
						1							Kepiro et al. 2008
	1	1			2					1		1	Hu et al. 2011
			1		1								Mei et al. 2012
			1				1						Zhang et al. 2013
			1	1	1	1		1				1	Qiu et al. 2015
						2	1	2					Li et al. 2016
	1												Zhang et al. 2016
		1				1	1				1		Fang et al. 2018
								2				1	Chen et al. 2021
		1		1			1			1	1		Yao et al. 2023

BRR: brown rice rate; MRR: milled rice rate; HMRR: head milled rice rate.
