# Supplementary material for: Identifying the Quantitative Trait Locus and Candidate Genes of Traits Related to Milling Quality in Rice via a Genome-Wide Association Study
Source: Plants (Basel). 2024 May 11;13(10):1324. doi: 10.3390/plants13101324 (PMC11124788; doi:10.3390/plants13101324)
Supplement: Supplementary file 1 [file plants-13-01324-s001.zip › Table S10.pdf]

Table S10. The sequences of primers used for the qRT-PCR.

Gene name	Forward primer sequence (5’-3’)	Reverse primer sequence (5’-3’)
UBQ	ACCCTGGCTGACTACAACATC	AGTTGACAGCCCTAGGGTG
LOC_Os05g08350	GTACTCGGAGTCACTGGCAAA	GTGGACCTTGAAGCACACTG
