# Supplementary material for: Identifying the Quantitative Trait Locus and Candidate Genes of Traits Related to Milling Quality in Rice via a Genome-Wide Association Study
Source: Plants (Basel). 2024 May 11;13(10):1324. doi: 10.3390/plants13101324 (PMC11124788; doi:10.3390/plants13101324)
Supplement: Supplementary file 1 [file plants-13-01324-s001.zip › Table S2.pdf]

Table S2. The results of the two-way analysis of variance for traits related to rice milling quality.

Traits	Source of variation	df	SS	MS	F-value	$F_{0.05}$	$F_{0.01}$
BRR /%	Genotypes	172	3569.3267	20.7519	17.2055**	1.31	1.52
	Environments	1	2.0632	2.0632	1.7106	3.90	6.81
MRR /%	Genotypes	172	2890.8088	16.8070	50.3185**	1.31	1.52
	Environments	1	1.6189	1.6189	4.8468*	3.90	6.81
HMRR /%	Genotypes	172	21665.9731	125.9650	10.5196**	1.34	1.52
	Environments	1	10.6719	10.6719	0.8912	3.90	6.81

df, degrees of freedom; SS, sum of squares; MS, mean square.* Significant differences at $P < 0.05$. ** Significant differences at $P < 0.01$. BRR, brown rice rate. MRR, milled rice rate. HMRR, head milled rice rate.
