# Supplementary material for: Identifying the Quantitative Trait Locus and Candidate Genes of Traits Related to Milling Quality in Rice via a Genome-Wide Association Study
Source: Plants (Basel). 2024 May 11;13(10):1324. doi: 10.3390/plants13101324 (PMC11124788; doi:10.3390/plants13101324)
Supplement: Supplementary file 1 [file plants-13-01324-s001.zip › Table S4.pdf]

Table S4. Candidate gene annotation in the region 4.43–4.57 Mb associated with the brown rice recovery.

Number	MSU ID	Position	Annotation
1	LOC_Os05g08130	4,429,556-4,432,477	Retrotransposon protein, Ty3-gypsy subclass
2	LOC_Os05g08140	4,434,988-4,435,902	Retrotransposon protein, Ty3-gypsy subclass
3	LOC_Os05g08230	4,478,544-4,483,011	Expressed protein
4	LOC_Os05g08240	4,496,316-4,498,339	Expressed protein
5	LOC_Os05g08250	4,501,034-4,503,616	Retrotransposon protein
6	LOC_Os05g08260	4,505,175-4,506,323	Retrotransposon protein
7	LOC_Os05g08310	4,535,682-4,542,064	Retrotransposon protein, Ty3-gypsy subclass
8	LOC_Os05g08320	4,543,967-4,545,215	Retrotransposon protein
9	LOC_Os05g08330	4,546,261-4,546,801	Hypothetical protein
10	LOC_Os05g08340	4,548,259-4,549,706	Expressed protein
11	LOC_Os05g08350	4,551,239-4,557,048	OsFBX162-F-box domain containing protein
12	LOC_Os05g08360	4,559,242-4,562,425	rRNA2-O-methyltransferase fibrillarin 2
13	LOC_Os09g38370	4,565,177-4,571,461	CESA1-cellulose synthase
