# Supplementary material for: Identifying the Quantitative Trait Locus and Candidate Genes of Traits Related to Milling Quality in Rice via a Genome-Wide Association Study
Source: Plants (Basel). 2024 May 11;13(10):1324. doi: 10.3390/plants13101324 (PMC11124788; doi:10.3390/plants13101324)
Supplement: Supplementary file 1 [file plants-13-01324-s001.zip › Table S5.pdf]

Table S5. The base information of F-box genes from five Gramineae plants.

No	Locus	Chr	Protein length	Start position	End position	Species
1	LOC_Os02g45320	2	297	27548399	27551592	*Oryza sativa*
2	LOC_Os02g56760	2	490	34798281	34801473	*Oryza sativa*
3	LOC_Os02g56840	2	297	34833867	34836156	*Oryza sativa*
4	LOC_Os03g02550	3	297	942694	945066	*Oryza sativa*
5	LOC_Os05g08350	5	554	4551239	4557048	*Oryza sativa*
6	LOC_Os08g05480	8	299	2918636	2921281	*Oryza sativa*
7	LOC_Os09g35680	9	287	20522699	20525471	*Oryza sativa*
8	LOC_Os12g03740	12	302	1514456	1519357	*Oryza sativa*
9	NP_001149956	1	296	80510246	80512782	*Zea mays*
10	NP_001356559	2	306	16670131	16672742	*Zea mays*
11	AQK79384	6	261	36193242	36196809	*Zea mays*
12	NP_001132379	7	296	141630967	141633481	*Zea mays*
13	NP_001152070	10	307	81194843	81197151	*Zea mays*
14	AQK45442	10	289	136725465	136728946	*Zea mays*
15	EER99217	2	299	65395929	65396047	*Sorghum bicolor*
16	XP_004962938	3	454	45918402	45921032	*Sorghum bicolor*
17	KXG31015	4	301	62578040	62582171	*Sorghum bicolor*
18	KXG31332	4	305	67228426	67231271	*Sorghum bicolor*
19	KXG31333	4	304	67232589	67234186	*Sorghum bicolor*
20	EES05996	4	279	67234844	67237079	*Sorghum bicolor*
21	KXG28410	5	270	52009210	52014439	*Sorghum bicolor*
22	EES11284	6	313	53935224	53938399	*Sorghum bicolor*
23	XP_002443222	8	402	49101599	49102807	*Sorghum bicolor*
24	KAG0520912	8	402	55352854	55354639	*Sorghum bicolor*
25	RCV07745	1	300	34364362	34367903	*Setaria italica*
26	RCV08867	1	292	41109880	41111682	*Setaria italica*
27	RCV12595	2	297	37863079	37865857	*Setaria italica*
28	RCV46890	9	287	58031101	58034338	*Setaria italica*
29	KQK19439	1	270	47064624	47071703	*Brachypodium distachyon*
30	KQK19441	1	252	47073892	47075067	*Brachypodium distachyon*
31	PNT76451	1	268	47079074	47080843	*Brachypodium distachyon*
32	PNT66655	3	303	13503351	13509187	*Brachypodium distachyon*
33	KQJ95438	3	295	15360409	15366773	*Brachypodium distachyon*
34	KQK01296	3	338	55122276	55124894	*Brachypodium distachyon*
35	KQK01300	3	305	55151560	55152842	*Brachypodium distachyon*
36	KQK01301	3	297	55154078	55156457	*Brachypodium distachyon*
37	KQJ91121	4	291	40968276	40970919	*Brachypodium distachyon*
