# Supplementary material for: Identifying the Quantitative Trait Locus and Candidate Genes of Traits Related to Milling Quality in Rice via a Genome-Wide Association Study
Source: Plants (Basel). 2024 May 11;13(10):1324. doi: 10.3390/plants13101324 (PMC11124788; doi:10.3390/plants13101324)
Supplement: Supplementary file 1 [file plants-13-01324-s001.zip › Table S6.pdf]

Table S6. The QTLs detected in this study overlapped with the QTLs/genes reported previously.

QTL name	Chr.	Position range /bp	Known QTL[a]/ association locus[a]	Flanking region /bp[a]	Reference
qBRR5.1	5	742,718-942,718	*qBR-5*	264,087-1,861,366	Weng et al. 2007
qMRR11	11	3,119,894-3,319,894	*qMR-11*	2,449,321-3,812,236	Weng et al. 2007
qHMRR2	2	33,924,415-34,124,415	*qHR2*	32,774,365-35,272,373	Hu et al.2011
				26,934,304-28,704,796	Tan et al. 2001
qHMRR5	5	27,851,340-28,051,340	*mr5*	22,671,210-33,689,061	Aluko et al. 2004
			qMR-5b	22,555,884-28,704,796	Weng et al. 2007
qHMRR11	11	24,733,345-24,933,345	*qHR11*	19,840,132-28,281,693	Yao et al. 2023

[a]The gene name, QTL name and the physical position (bp) was inferred from the database of Gramene website (http://www.gramene.org/markers/), BLAST (http://blast.ncbi.nlm.nih.gov/Blast.cgi) and the China Rice Data Center database (http://www.ricedata.cn/gene/list/1499.htm).
